# Supplementary material for: 2N+4-rule and an atlas of bulk optical resonances of zigzag graphene nanoribbons
Source: Nat Commun. 2020 Jan 3;11:82. doi: 10.1038/s41467-019-13728-8 (PMC6941967; doi:10.1038/s41467-019-13728-8)
Supplement: Supplementary file 2 — Supplementary Information [file 41467_2019_13728_MOESM2_ESM.pdf]

Supplementary Information

for

2N+4-rule and an atlas of bulk optical resonances of zigzag graphene nanoribbons

Renebeth B. Payod,[1] Davide Grassano,[2] Gil Nonato C. Santos,[1] Dmitry I. Levshov,[3,6] Olivia Pulci,[2] and Vasil A. Saroka*,[4,5]

1 Physics Department, De La Salle University, 2401 Taft Avenue, 0922 Manila, Philippines

2 Department of Physics, and INFN, University of Rome Tor Vergata, Via della Ricerca Scientifica 1, Rome, I-00133 Italy

3 Faculty of Physics, Southern Federal University, 5 Zorge Str., Rostov-on-Don, 344090, Russia

4 Center for Quantum Spintronics, Department of Physics, Norwegian University of Science and Technology, NO-7491, Trondheim, Norway

5 Institute for Nuclear Problems, Belarusian State University, Bobruiskaya 11, 220030 Minsk, Belarus

6 present address: Physics Department, University of Antwerp, Universiteitsplein 1, B-2610 Antwerp, Belgium

E-mail: vasil.saroka@ntnu.no

Supplementary Discussion

Although the single electron picture is generally a crude model, majority of the measurements on individual SWCNT[1,2] and SWCNT suspensions[3] testify that the many-body effects can be incorporated into a single-particle tight-binding model (TBM) by rescaling and increasing the number of hopping and overlapping integrals. In contrast to most of the literature on zigzag nanoribbons, the physics of the edge states[4–9] is not the focus of our study. Therefore, in our first principle calculations for the ZGNR, the energy bands are not spin polarized. These calculations are also performed on non-relaxed nanostructures. As shown by both Son et. al.[10] and Yang et. al.,[11] accounting the spins and C-C bond relaxation in the band structure calculations opens an energy gap between the pair of flat edge states in the narrow ZGNRs but gives little effect to the bulk states, which are of our primary interest. Similar arguments apply to carbon nanotubes (see discussion of the curvature effects and intrinsic strain in Supplementary References 12,13).

Supplementary Note 1. Tight-binding parameters goodness

Before performing TBM calculations, we checked that the fitted tight-binding parameters provide a reasonable approximation to the DFT band structure in the region around $k = 2\pi/3$, which gives a major contribution into the optical absorption peaks of ZGNRs.[14] As shown in Supplementary Figure 1, the averaged set of parameters provides a good description not only to the four above mentioned ZGNRs but also to the narrower ZGNR(5) and to the significantly wider ZGNR(20).

Calculating the DFT band structures of the carbon nanotubes, we noticed that these data were perfectly described by the set of 7 tight-binding parameters reported by Reich et al.[15] (Reich2002) TBM; the used parameters are given in Table 1 of the main text. Supplementary

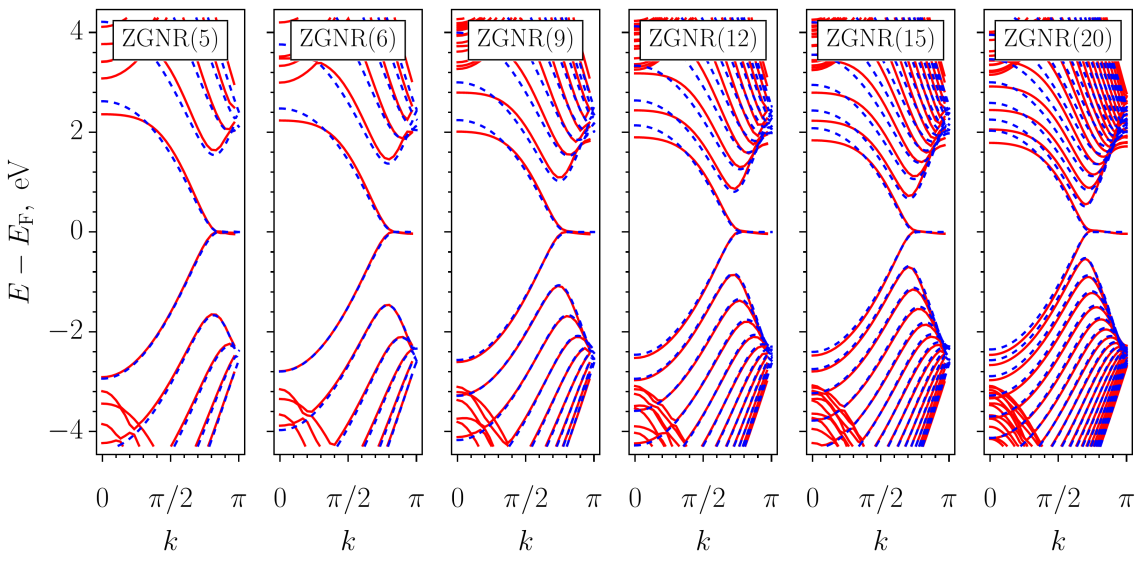

Supplementary Figure 1. Goodness of the fitted TBM parameters for ZGNRs. Comparison between the ZGNR bandstructures calculated with DFT Quantum Espresso package (red solid) and ZGNRs(av.) TBM parameters given in Table 1 of the main text (blue dashed).

Figure 2 shows that these parameters describe the DFT bandstructures with high precision for both narrow and wide diameter armchair SWCNTs.

Supplementary Note 2. Optical absorption spectra

In this Supplementary Note, we provide optical absorption spectra plots for all considered structures and visualize the results of the peak structure analysis carried out for zigzag nanoribbons and armchair nanotubes within different tight-binding models. For both nanostructures in question, we first performed calculations with a single parameter TBM with $t_1 = 3.12$ eV as suggested by Partoens et al.[16] (Partoens2006). Then, to reveal the effect of the higher order nearest-neighbours hopping and overlapping integrals, we calculated the optical absorption spectra with TBMs fitted to DFT results.

In Supplementary Figure 3, we present the results of our calculations in Partoens2006 TBM[16] for all ribbons with the width index $w = 2, 3, \ldots, 60$. The index is related to the width of the zigzag ribbon as $W = \sqrt{3}aw/2$, where $a = 2.46$ Å. For each ribbon, we performed two runs, namely, one for full absorption spectra including all edge-to-bulk

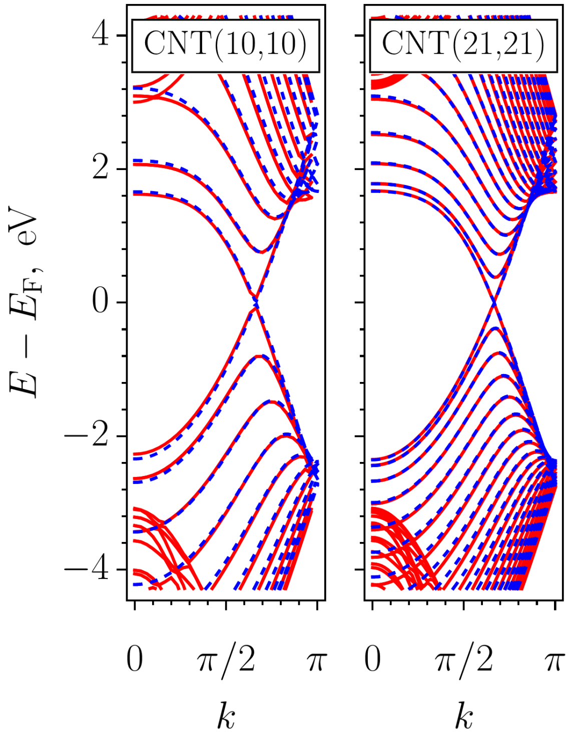

Supplementary Figure 2. Goodness of the fitted TBM parameters for armchair SWCNTs. Comparison between armchair SWCNT bandstructures calculated with DFT Quantum Espresso package (red solid) and 7 TBM parameters reported by Reich et al.[15] (Reich2002) (blue dashed).

.

and bulk-bulk transitions and another run for the reduced absorption spectra that include only bulk-bulk interband transitions. The significant optical absorption resonances were extracted by our algorithm (see Methods section) with $x = 8\%$ and $dE = 0.07$ eV. The results obtained in similar calculations within the fitted TBM ZGNRs(av) (see Table 1 of the main text) are presented in Supplementary Figure 4. The resonances highlighted in this case were extracted by the algorithm with $x = 4\%$ and $dE = 0.1$ eV. It is seen from both Supplementary Figures that in each case the algorithm indeed identified all major optical resonances. Another interesting observation is that the contribution of the edge states to the absorption significantly decreases for increasing ribbon width so that for $w \gtrsim 35$ only lowest in energy absorption resonance retains the pure edge-to-bulk state nature.

The optical absorption spectra of single-wall armchair carbon nanotubes are presented in Supplementary Figures 5 and 6. These Supplementary Figures also compare the armchair SWCNTs absorption spectra with the bulk spectra of ZGNRs with width index $w = n - 1$. The bulk spectra of ZGNRs in Supplementary Figures 5 and 6 are normalized by the maximum intensity in the tube spectrum. The armchair SWCNT diameter index n ranges from 4 to 60 and defined as $n = \pi d/(\sqrt{3}a)$, where d is the tube diameter and $a = 2.46$ Å. The red circles in Supplementary Figures 5 and 6 mark those intensity peaks that were selected by our algorithm with $x = 19\%$ and $dE = 0.1$ eV for Partoens2006 TBM (Supplementary Figure 5) and $x = 7$ % and $dE = 0.05$ eV for Reich2002 TBM (Supplementary Figure 6). Again it is seen that the specified parameters allow one to extract all major resonances. Unfortunately, we were unable to skip with the algorithm the second lowest in energy peak in the panel for $n = 28$ of the Supplementary Figure 5. Although this peak arises in the spectrum due to the numerical reasons, we left it in the data used for LCC and AC maps. However, we excluded this peak from the data used in the fitting of the linear transformation parameters for the atlas ZGNR optical resonances.

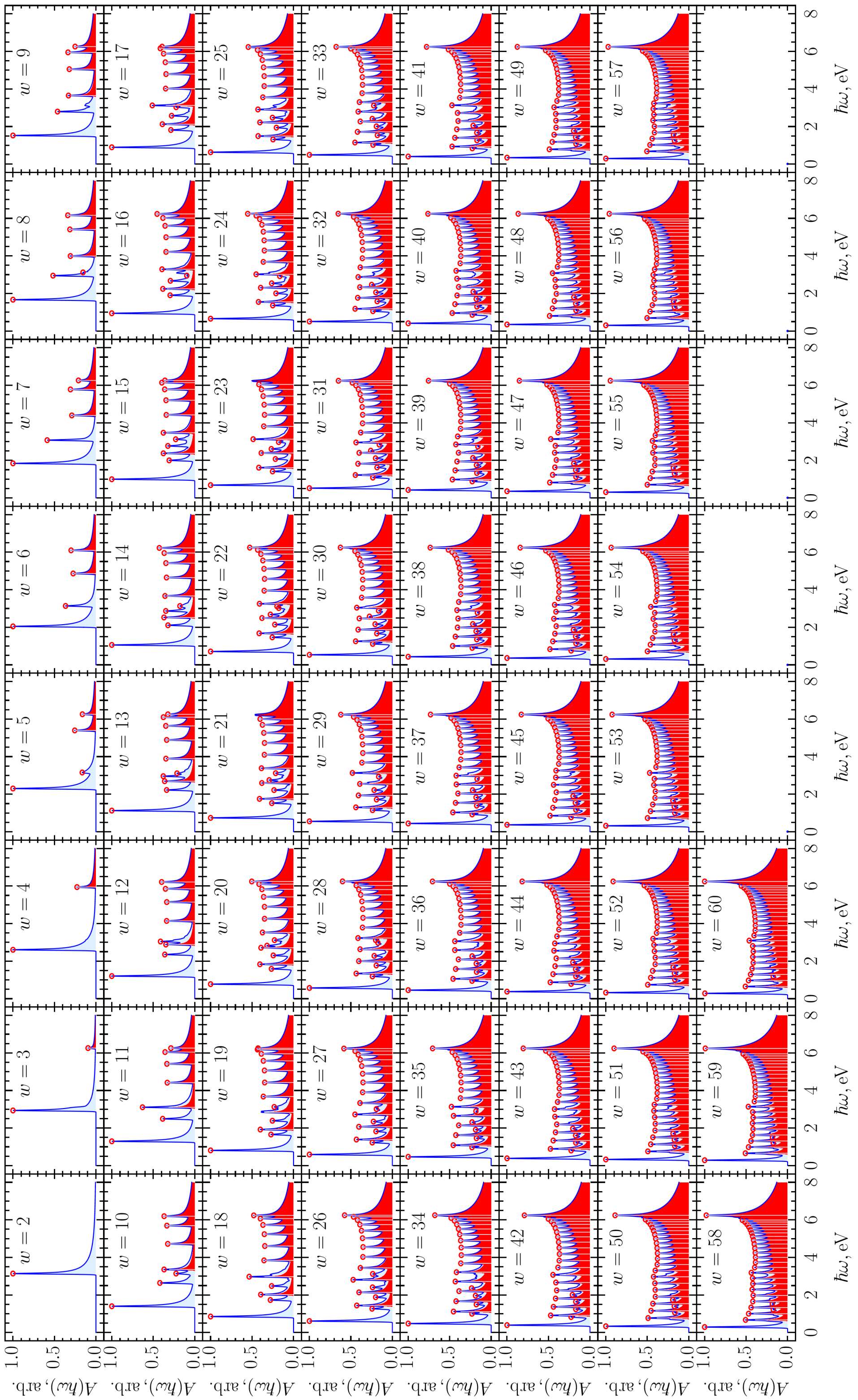

Supplementary Figure 3. Absorption spectra of ZGNRs in Partoens2006 TBM. The full (blue solid) and bulk (red filled) spectra are given for each panel labeled with the width index w. Red circles denote position of the extracted peaks. Similar positions extracted from the bulk absorption spectra are denoted by white half transparent lines.

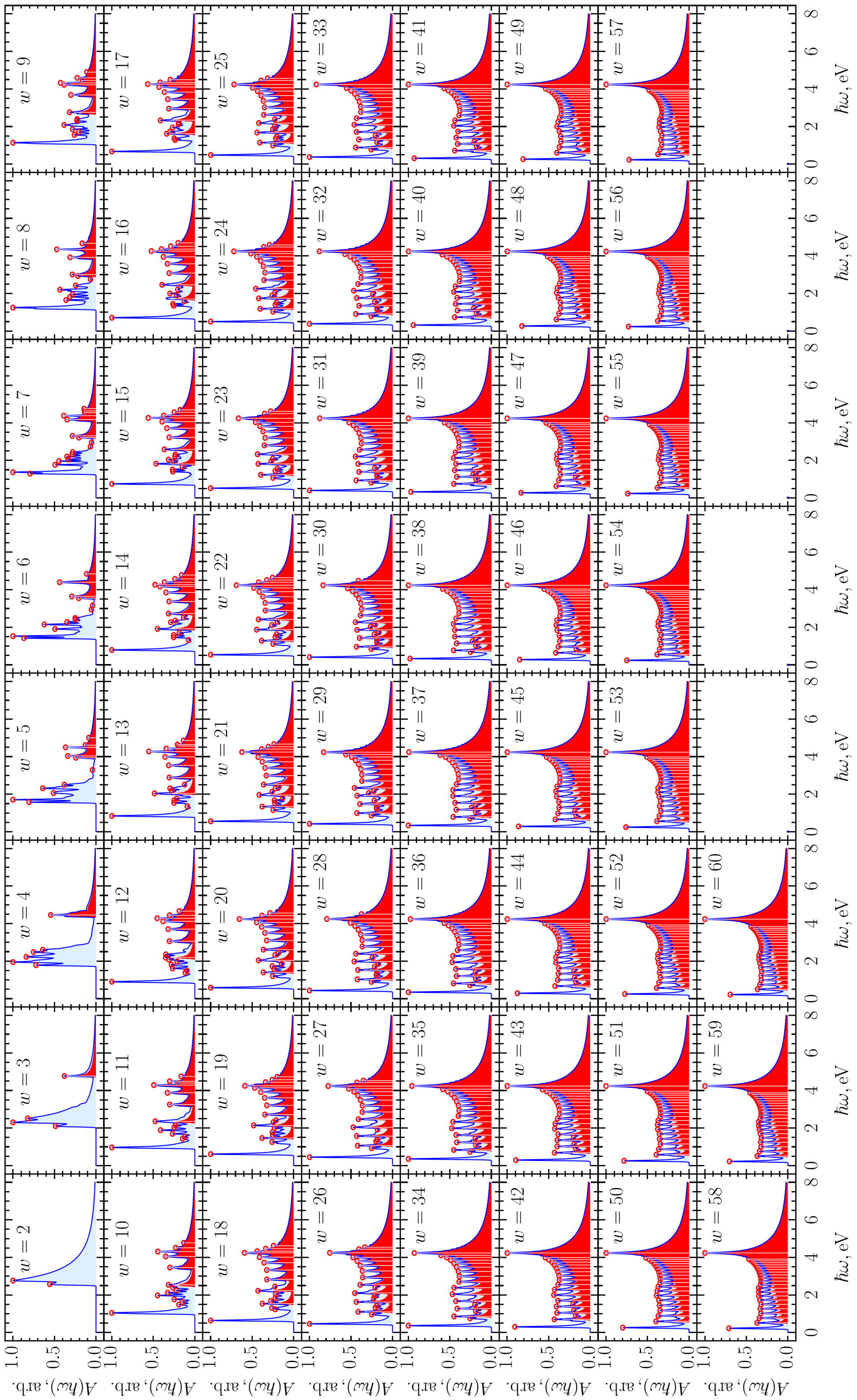

Supplementary Figure 4. Absorption spectra of ZGNRs in the fitted TBM. Same as Supplementary Figure 3, but for the fitted 6 parameter TBM ZGNR(av.) presented in Table 1 of the main text.

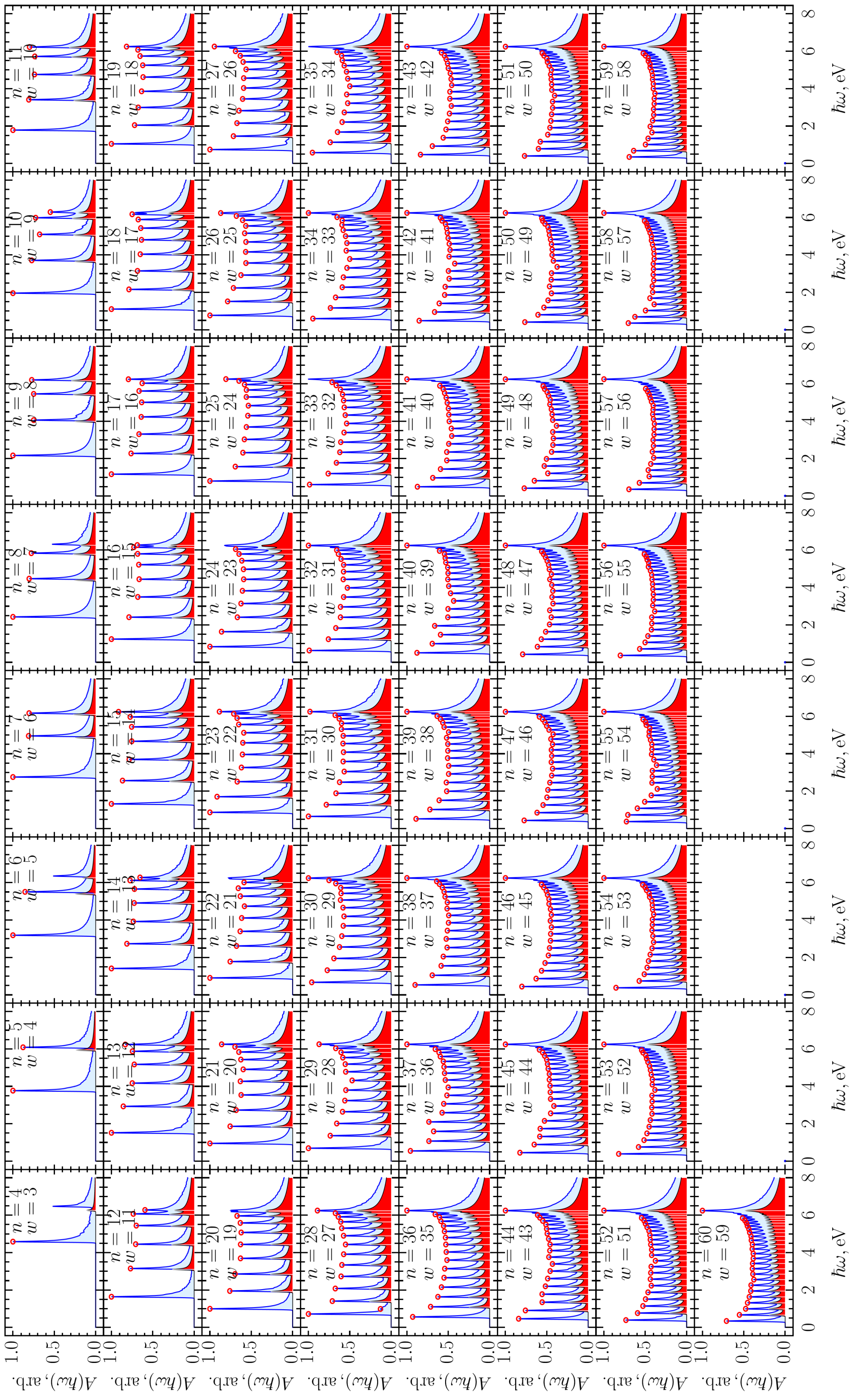

Supplementary Figure 5. Absorption spectra of armchair SWCNTs in Partoens2006 TBM. The absorption spectra of armchair SWCNTs with diameter index n (blue solid filled) are overlayed with bulk optical absorption spectra of ZGNRs (red filled). The position of absorption peaks extracted by our algorithm for the tube and bulk ribbons spectra are presented by red circles and white vertical lines, respectively.

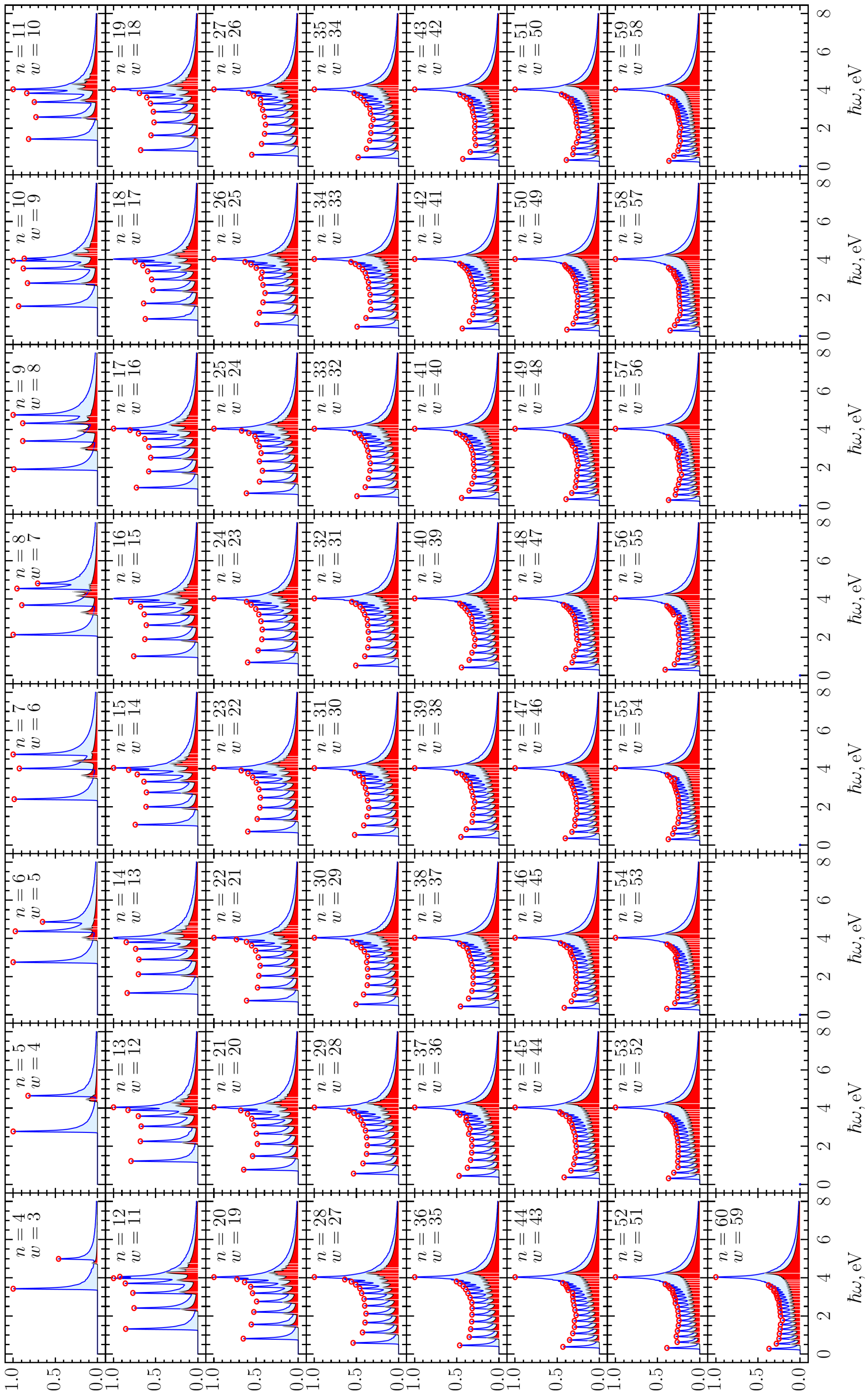

Supplementary Figure 6. Absorption spectra of armchair SWCNTs in Reich2002 TBM. Same as Supplementary Figure 5, but for TBM developed by Reich et al.[15]

Supplementary Note 3. Mapping armchair SWCNT transitions to ZGNR transitions

In this Supplementary Note, we show that armchair SWCNT optical resonances can be mapped to the bulk optical resonances of ZGNRs. The high values of the linear correlation and alignment coefficients suggest that this transformation is linear:

$$E_{\mathrm{ZGNR}} = a_{\mathrm{M}}(n) E_{\mathrm{aSWCNT}} + b_{\mathrm{M}}(n) \quad (1)$$

where 'M' stands for the model, i.e. 'P' for Partoens2006 or 'R' for Reich2002, and $a_{\mathrm{M}}(n)$ and $b_{\mathrm{M}}(n)$ do not depend on the tube transition resonance energy and are approximately equal to 1 and 0, respectively.

In order to verify the above conclusion, we fit all tube-ribbon pairs related by $n = w + 1$ in the range $n = 6, 7, \ldots, 60$; as one can see from Supplementary Figures 5 and 6, structures with $n < 6$ provide too few points for their reliable fitting. The collected fitting coefficients $a_{\mathrm{M}}(n)$ and $b_{\mathrm{M}}(n)$ for Partoens2006 and Reich2002 TBMs are shown in Supplementary Figure 7. Indeed, in the first approximation $a_{\mathrm{M}}(n) \approx 1$ and $b_{\mathrm{M}}(n) \approx 0$ with both having a slight transverse size dependence. The transverse size dependence can be described as $a_{\mathrm{M}}(n) = (\xi_{\mathrm{M},1}/n) + \zeta_{\mathrm{M},2}$ and $b_{\mathrm{M}}(n) = (\xi_{\mathrm{M},3}/n) + \zeta_{\mathrm{M},4}$, where $\xi_{\mathrm{M},1(3)}$ and $\zeta_{\mathrm{M},2(4)}$ are new parameters to be determined. The results of $\xi_{\mathrm{M},1(3)}$ and $\zeta_{\mathrm{M},2(4)}$ fitting are given in Supplementary Table 1. These parameters are obtained by fitting only the points $n \geq 10$, which represent the regular part of the plots in Supplementary Figure 7. This means that the mapping should be more precise for ribbons wider than 1.9 nm. In Partoens2006 TBM, the point $n = 28$ was excluded from fitting since it represents a spurious low intensity peak that can be seen in the corresponding panel of Supplementary Figure 5. This spurious peak also causes distortion to the alignment coefficient map at $n = 28$ presented in Figure 3 of the main text.

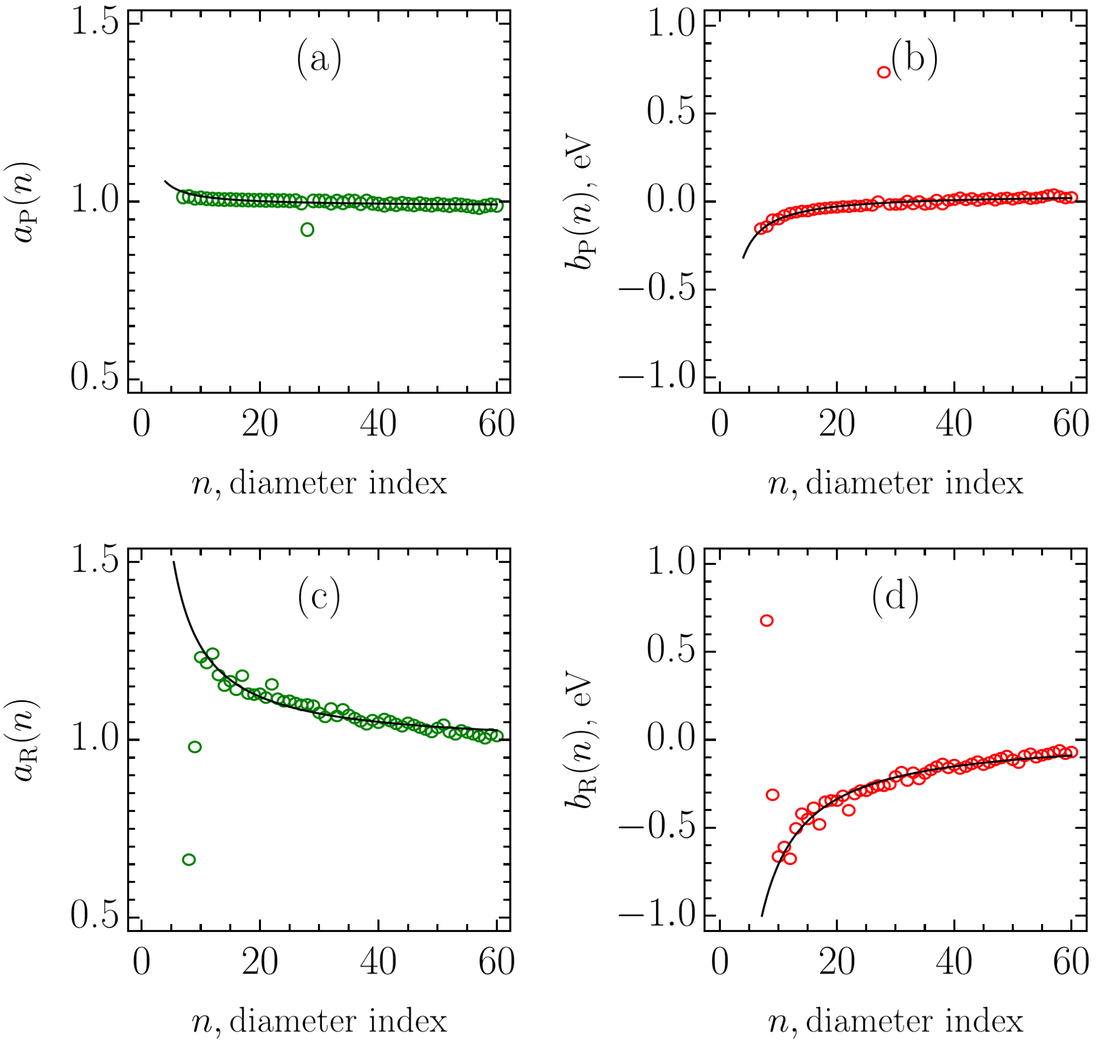

Supplementary Figure 7. Linear transformation coefficients. The fitted parameters of the linear tube-ribbon mapping as functions of the armchair SWCNT diameter: (a-b) the nearest-neighbor Partoens2006 TBM,[16] and (c-d) Reich et al. TBM[15] Black curves present fitting of the diameter dependence with $\xi/x+\zeta$ functional form.

Supplementary Table 1. The fitted parameters for $a_{\mathrm{M}}(n)$ and $b_{\mathrm{M}}(n)$ coefficients.

	$\xi_{\mathrm{M},1}$	$\zeta_{\mathrm{M},2}$	$\xi_{\mathrm{M},3}$, eV	$\zeta_{\mathrm{M},4}$, eV
Partoens2006 (P)	0.28	0.99	−1.45	0.04
Reich2002 (R)	2.83	0.98	−7.42	0.03

In Supplementary Figure 8, we compare the optical resonance dependencies on the nanostructure transverse size in different models. In panels (a) and (b), one can see that the linear mapping with the parameters from Supplementary Table 1 shows a good agreement with the initial numerical data thereby supporting the linear functional form connecting tube and bulk-bulk ribbon optical resonances. Since linear mapping is valid for several models, we assume that it can be applied to experimentally measured and interpolated energies of the optical resonances provided by Liu et al. for armchair SWCNTs in Supplementary Reference 2. Therefore, it is interesting to compare our numerical data with the interpolated experimental data from other studies. In panel (c), it is seen that Reich2002 TBM underestimates the resonance energy and overestimates the number of optical resonances for a given tube, but it provides a correct transverse size dependence for the resonances, which results in a perfect match between several optical resonance branches. Since the coefficients in Supplementary Table 1 depends only on the transverse size of the tubes and not on the energies of optical resonances, they can be used in Supplementary Equation 1 to map the armchair SWCNTs resonances given by Liu et al. interpolating formula.[2] In panel (d), we also notice that the size dependence in question is very similar to that given by the analytic expression for the separation between the van Hove singularities of the bands connected by allowed optical transitions: $2t_1 \sin[\pi p/n]$, where t_1 is the nearest neighbor hopping integral effectively set to 2 eV and p is an integer labeling the optical resonances. In fact, we have checked that introducing $p \rightarrow \xi p + \zeta$, where ξ and ζ are some new fitting parameters, provides a reasonable fit for the armchair SWCNT resonances given by the Liu et al. formula.[2] From a practical point of view, however, it does not matter how the energies inserted into Supplementary Equation 1 were obtained, therefore we recommend the use of the original and more general interpolating formula. In summary, the presented results provide a new insight into the functional forms that can be used for the interpolation of the experimental data and extend the applicability of the Liu et al. interpolation formula[2] to the case of zigzag graphene nanoribbons.

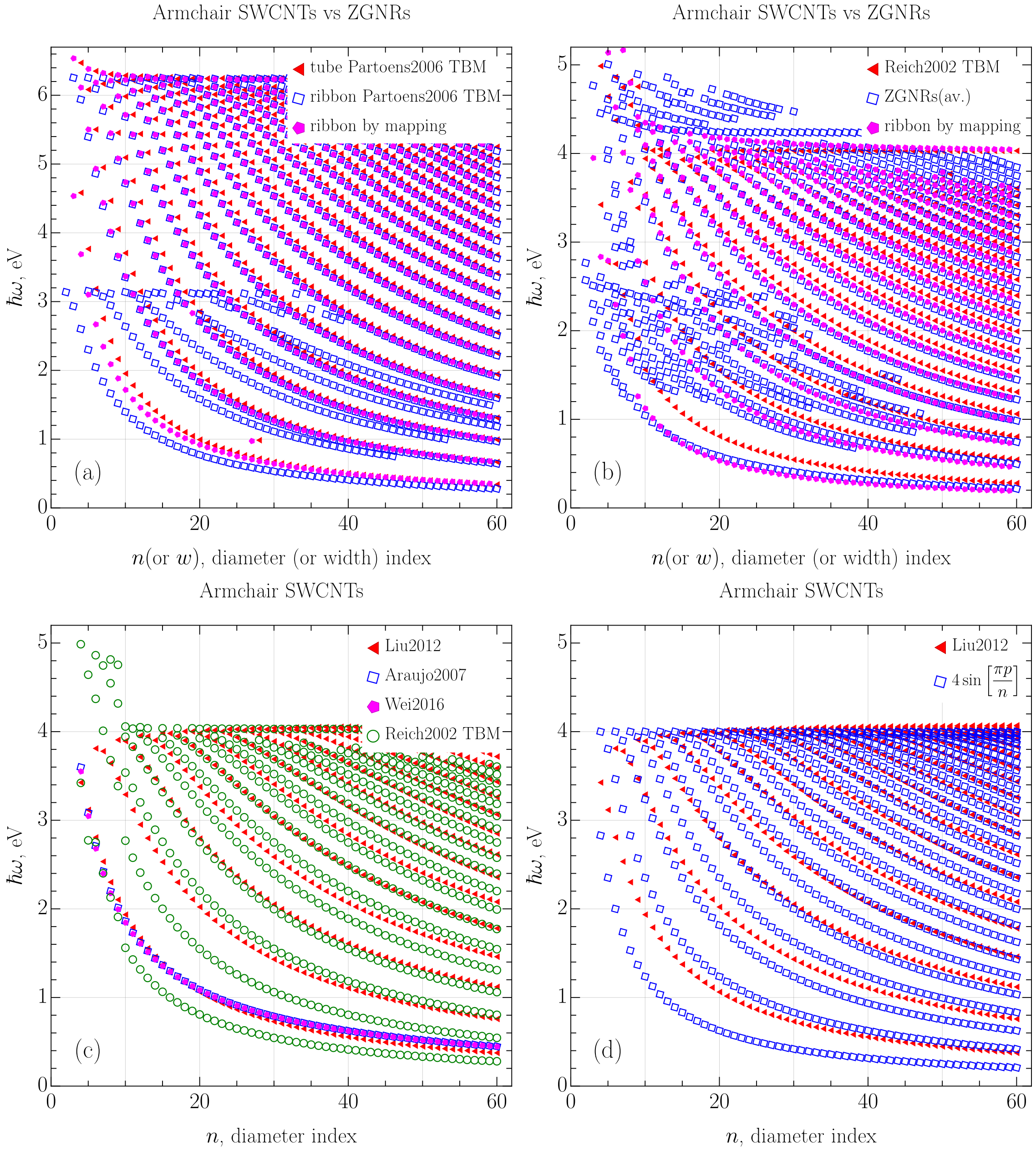

Supplementary Figure 8. ZGNR and armchair SWCNT absorption resonances. (a-b) The comparison between Kataura plots of ZGNRs and armchair SWCNTs in nearest-neighbor Partoens2006 TBM[16] and non-orthogonal third order nearest neighbour Reich2002 TBM.[15] (c-d) The comparison between Kataura plots for armchair SWCNTs in numerical models and experimentally obtained interpolating formulas: Liu2012,[2] Araujo2007,[17] Wei2016;[18] $4\sin[\pi p/n]$ is Equation (46) from Supplementary Reference 14, where nearest neighbour hopping intergral is set to 2 eV.

Supplementary References

(1) Michel, T.; Paillet, M.; Meyer, J. C.; Popov, V. N.; Henrard, L.; Sauvajol, J.-L. E33 and E44 optical transitions in semiconducting single-walled carbon nanotubes: Electron diffraction and Raman experiment. *Phys. Rev. B* **2007**, *75*, 155432.

(2) Liu, K.; Deslippe, J.; Xiao, F.; Capaz, R. B.; Hong, X.; Aloni, S.; Zettl, A.; Wang, W.; Bai, X.; Louie, S. G.; Wang, E.; Wang, F. An atlas of carbon nanotube optical transitions. *Nat. Nanotechnol.* **2012**, *7*, 325–329.

(3) Weisman, R. B.; Bachilo, S. M. Dependence of optical transition energies on structure for single-walled carbon nanotubes in aqueous suspension: An empirical Kataura plot. *Nano Lett.* **2003**, *3*, 1235–1238.

(4) Fujita, M.; Wakabayashi, K.; Nakada, K.; Kusakabe, K. Peculiar localized state at zigzag graphite edge. *J. Phys. Soc. Japan* **1996**, *65*, 1920–1923.

(5) Kane, C. L.; Mele, E. J. Quantum spin Hall effect in graphene. *Phys. Rev. Lett.* **2005**, *95*, 226801.

(6) Magda, G. Z.; Jin, X.; Hagymási, I.; Vancsó, P.; Osváth, Z.; Nemes-Incze, P.; Hwang, C.; Biró, L. P.; Tapasztó, L. Room-temperature magnetic order on zigzag edges of narrow graphene nanoribbons. *Nature* **2014**, *514*, 608–611.

(7) Ruffieux, P.; Wang, S.; Yang, B.; Sánchez-Sánchez, C.; Liu, J.; Dienel, T.; Talirz, L.; Shinde, P.; Pignedoli, C. A.; Passerone, D.; Dumslaff, T.; Feng, X.; Müllen, K.; Fasel, R. On-surface synthesis of graphene nanoribbons with zigzag edge topology. *Nature* **2016**, *531*, 489–492.

(8) Su, X.; Xue, Z.; Li, G.; Yu, P. Edge state engineering of graphene nanoribbons. *Nano Lett.* **2018**, *18*, 5744–5751.

(9) Sichau, J.; Prada, M.; Anlauf, T.; Lyon, T. J.; Bosnjak, B.; Tiemann, L.; Blick, R. H. Resonance microwave measurements of an intrinsic spin-orbit coupling gap in graphene: A possible indication of a topological state. *Phys. Rev. Lett.* **2019**, *122*, 046403.

(10) Son, Y.-W.; Cohen, M. L.; Louie, S. G. Energy gaps in graphene nanoribbons. *Phys. Rev. Lett.* **2006**, *97*, 216803.

(11) Yang, L.; Park, C.-H.; Son, Y.-W.; Cohen, M. L.; Louie, S. G. Quasiparticle energies and band gaps in graphene nanoribbons. *Phys. Rev. Lett.* **2007**, *99*, 186801.

(12) Portnoi, M. E.; Saroka, V. A.; Hartmann, R. R.; Kibis, O. V. Terahertz applications of carbon nanotubes and graphene nanoribbons. 2015 IEEE Comput. Soc. Annu. Symp. VLSI. 2015; pp 456–459.

(13) Hartmann, R. R.; Saroka, V. A.; Portnoi, M. E. Interband transitions in narrow-gap carbon nanotubes and graphene nanoribbons. *J. Appl. Phys.* **2019**, *125*, 151607.

(14) Saroka, V. A.; Shuba, M. V.; Portnoi, M. E. Optical selection rules of zigzag graphene nanoribbons. *Phys. Rev. B* **2017**, *95*, 155438.

(15) Reich, S.; Maultzsch, J.; Thomsen, C.; Ordejón, P. Tight-binding description of graphene. *Phys. Rev. B* **2002**, *66*, 035412.

(16) Partoens, B.; Peeters, F. M. From graphene to graphite: Electronic structure around the K point. *Phys. Rev. B* **2006**, *74*, 075404.

(17) Araujo, P. T.; Doorn, S. K.; Kilina, S.; Tretiak, S.; Einarsson, E.; Maruyama, S.; Chacham, H.; Pimenta, M. A.; Jorio, A. Third and fourth optical transitions in semiconducting carbon nanotubes. *Phys. Rev. Lett.* **2007**, *98*, 067401.

(18) Wei, X.; Tanaka, T.; Yomogida, Y.; Sato, N.; Saito, R.; Kataura, H. Experimental determination of excitonic band structures of single-walled carbon nanotubes using circular dichroism spectra. *Nat. Commun.* **2016**, *7*, 12899.
